# The Impact of Creative Writing on Patients With Chronic Pain

**DOI:** 10.7759/cureus.112460

**Published:** 2026-07-11

**Authors:** Konstantinos Prassas, Nikolas Dovrolis, Aikaterini Arvaniti, Aikaterini Terzoudi, Pelagia Chloropoulou

**Affiliations:** 1 Medicine, Democritus University of Thrace, Alexandroupoli, GRC; 2 Psychiatry, Democritus University of Thrace, Alexandroupoli, GRC; 3 Neurology, Democritus University of Thrace, Alexandroupoli, GRC; 4 Anesthesiology, Democritus University of Thrace, Alexandroupoli, GRC

**Keywords:** anxiety, chronic pain, creative writing, depression, palliative care

## Abstract

Aims and objectives

Creative writing has already been utilized within the context of care and rehabilitation of patients suffering from various mental and physical conditions, yielding positive outcomes. The aim of this study is to examine whether and how the psychosomatic state of chronic pain patients is affected by their engagement with creative writing.

Methodological design

The study employed a non-randomized design and used a longitudinal controlled quasi-experimental design with two parallel groups assessed at three timepoints: pre-intervention (T1), mid-intervention (T2), and post-intervention (T3).

Ethical issues and approval

Participants provided written informed consent, and the study was approved by the Scientific Council of the University General Hospital of Alexandroupolis (approval number: 50535/18-10-2023).

Research methods and interventions

This controlled longitudinal quasi-experimental study included 120 adults with chronic pain, allocated to an intervention group (n = 60) or a control group (n = 60), and compared the effects of a creative writing intervention against a waitlist control on self-assessed pain severity, quality of life, anxiety, and self-rated health, assessed at three timepoints (T1 = baseline; T2 = mid-point; T3 = post-intervention).

Results

Over an observation period of two weeks, the control group showed near-zero change on every outcome: pain, anxiety, functional impairment, and health. The intervention group, by contrast, showed a progressive improvement in self-rated health and a meaningful decrease in anxiety, while pain severity and functional impairment remained broadly stable.

Conclusions

The intervention was associated with significant reductions in anxiety and depression and meaningful improvements in self-rated health by the end of the observation period. Pain severity itself did not change significantly at the group level, but the way pain related to other aspects of quality of life differed markedly between the two groups.

## Introduction

Chronic pain is defined as the pain experienced by a patient for a duration exceeding three months, and it affects various parts of the body, involving frequently the musculoskeletal system [1]. Causes vary from a previous injury to a chronic degenerative, neoplastic, or infiltrating disease, although occasionally no specific underlying cause is recognized. Each patient approaches chronic pain in their own unique way, determining its intensity and severity at largely different levels [2], which may be influenced, to a lesser or greater extent, by various biological, psychological, and social factors [3]. Importantly, chronic pain often leads to secondary disorders that affect the patient's psychosomatic state, thereby degrading their quality of life [4]. Ultimately, unlike acute forms, this chronic condition does not serve a protective function, but constitutes a distinct disease in its own right [5].

Creative writing is the composition of literary texts based on the author's imagination and experience [6]. The creator selects the textual genre they deem appropriate and attempts to present their story through written discourse [7]. Certainly, the creative dimension of this endeavor mandates free and, at times, unconventional and original expression, rendering it attractive and engaging for all [8]. The presentation and recording of stories through digital media can also be utilized when a potential creator finds it difficult or is reluctant to engage in the writing process. Digital storytelling can touch upon various fields, naturally including existential concerns and reflections [9].

Patients suffering from chronic pain often experience intense frustration, stemming from both the prolonged duration of the painful period and the perception that they are not receiving the appropriate medical care required to improve their health status [5]. To foster a closer bond between patients and medical/nursing staff, as well as to mitigate general patient dissatisfaction, creative/expressive writing has previously been employed [10]. The utilization of creative writing can contribute to a small but noticeable reduction in depression, anxiety, and stress, particularly when there is a short interval between the writing sessions performed by patients [11]. Furthermore, creative writing is already being utilized with positive outcomes within the context of care and rehabilitation for patients suffering from various mental and physical conditions [12]. Creative/expressive writing can also be utilized to reduce stress and improve the psychological well-being of healthcare professionals who often suffer from burnout [13].

The purpose of the present study is to examine whether, and to what extent, engagement with creative writing/digital storytelling can influence the health status of chronic pain sufferers and their general outlook on life, as has been observed in other patient groups [14].

## Materials and methods

Study design and ethical approval

The study used a longitudinal controlled quasi-experimental design with two parallel groups assessed at three timepoints: pre-intervention (T1), mid-intervention (T2), and post-intervention (T3). The analytic sample included 120 adults with chronic pain, with 60 participants in the intervention group on top of standard of care (SoC) and 60 participants in the SoC control group.

The study employed a non-randomized design. Group allocation was determined consecutively based on the timing of participant enrollment, namely, the first 60 participants were assigned to the control group, whereas the subsequent 60 participants were assigned to the intervention group. Meetings and collaboration with the patients of both groups took place at the pain clinic of the University General Hospital of Alexandroupolis (P.G.N.A.). The clinic and medical personnel remained unchanged throughout the course of the study. Both groups completed the same questionnaires at the same assessment intervals, producing 360 person-occasion observations. Participants provided written informed consent, and the study was approved by the Scientific Council of the University General Hospital of Alexandroupolis (approval number: 50535/18-10-2023).

Questionnaires

The intervention group was initially asked to complete two brief questionnaires: the Greek Brief Pain Inventory (GBPI) short form (Appendix A) and the Greek version of the EuroQol 5-Dimension 5-Level (EQ-5D-5L) health questionnaire (Appendix B) [15]. The members of the control group completed the same questionnaires as the intervention group at the corresponding time intervals (T1: baseline, T2: end of the first week, T3: end of the second week).

Intervention description

Among the intervention group, for one week, 45 participants recorded (in writing) and 15 narrated on a daily basis, for 15 minutes per day, their thoughts and feelings regarding their current health status and the chronic pain they experience. Upon completion of the first week, the experimental group re-completed the two aforementioned questionnaires. Then, during the second week of the intervention, the 45 participants recorded and the 15 narrated, again for 15 minutes daily, their thoughts and expectations for the future. At the end of this week, the participants completed the questionnaires for the third and final time, marking the conclusion of the intervention. The files generated through oral narration share similar content with those obtained through writing. Narration was chosen by elderly individuals participating in the intervention group, due to their difficulties with writing. The control group did not engage in any recording or narration of thoughts and emotions. However, they cooperated with the researcher at all three distinct timepoints of the study (T1: baseline, T2: end of the first week, T3: end of the second week) while completing the questionnaires.

Statistical analysis

Analyses were organized into primary, secondary, and exploratory blocks. Multiple analytical models were employed to account for baseline differences between the groups. The questionnaires administered helped in quantifying concepts like anxiety. The primary analysis tested group, time, and group-by-time effects on pain severity, anxiety/depression, functional impairment, medication effectiveness, and self-rated health using linear mixed-effects models with participant-level random intercepts. Models included age and sex as covariates; perceived medication effectiveness was included as a predictor or moderator where relevant. Pairwise post hoc contrasts were adjusted within each analytic block using the Holm correction. No global correction was applied across blocks because each block addressed a distinct analysis question, but exploratory analyses are interpreted as hypothesis-generating rather than confirmatory. Model diagnostics included residual-versus-fitted plots, quantile-quantile plots, Cook's distance, the Breusch-Pagan test for heteroskedasticity, and the Shapiro-Wilk test of residual normality. The alpha level was set at 0.05 throughout. Effect sizes are reported alongside significance tests [16]. All linear mixed models (LMMs) were evaluated diagnostically using residual-versus-fitted plots, quantile-quantile plots of residuals, Cook's distance, the Breusch-Pagan test for heteroskedasticity, and the Shapiro-Wilk test of residual normality. All analyses were conducted in R (version 4.4.0) (R Foundation for Statistical Computing, Vienna, Austria) [17].

Secondary analyses included T1-to-T3 paired change tests within each group, moderation models testing whether perceived medication effectiveness altered the potential association between pain severity and outcomes, and a multigroup mediation/path model testing whether anxiety/depression statistically mediated the association between pain severity and functional impairment. Normality of paired difference scores was evaluated before choosing paired t-tests or Wilcoxon signed-rank tests. Exploratory analyses included correlation-structure comparisons using Fisher r-to-z tests, Spearman correlations with bootstrap confidence intervals, participant-level trajectory slopes, k-means clustering of response profiles, ordinal logistic sensitivity models for the EQ-5D anxiety/depression outcome, a piecewise mixed model for timing of anxiety change, and models testing whether anxiety benefit differed by age or sex.

## Results

Baseline characteristics

Baseline demographic and clinical characteristics are shown in Table 1.

**Table 1:** Baseline demographic and clinical characteristics of the study participants. Data are presented as median (IQR) for continuous variables and n (%) for categorical variables Age was non-normally distributed in both groups (Shapiro-Wilk: intervention (W = 0.953; p = 0.021) and control (W = 0.926; p = 0.001)) and was compared using the MWU. Group-level chi-squared tests compare the full distribution across all sub-categories within each characteristic. Pain location was tested with abdominal pain and gastroesophageal reflux combined into a single "Other" category to avoid expected cell frequencies below 5. The primary diagnosis chi-squared test includes cells with expected frequency <5 (minimum expected = 1.0) due to the absence of Crohn's disease and obesity in the control group. The result should be interpreted with caution; a Fisher exact-based permutation test would be more appropriate for confirmatory inference. Polypharmacy: intervention group: 31 monotherapy (51.7%) and 29 combination therapy (48.3%); control group: 39 monotherapy (65.0%) and 21 combination therapy (35.0%). Individual drug-level Fisher's exact tests treat each drug as a binary exposure (prescribed vs. not prescribed). Tests are independent, and p-values are unadjusted for multiple comparisons; results should be interpreted as exploratory. Patients on combination therapy may appear in more than one drug row; frequencies are not mutually exclusive and do not sum to group totals. Other agents (intervention, n = 15 patients): alprazolam (n = 2), ustekinumab (n = 2), lansoprazole (n = 2), nimesulide (n = 2), methylprednisolone (n = 2), lorazepam (n = 1), carbamazepine (n = 1), diazepam (n = 1), mirtazapine (n = 1), duloxetine (n = 1). Other agents (control, n = 5 patients): naproxen/orphenadrine (n = 2), alprazolam (n = 2), ibuprofen (n = 1). Notable between-group imbalances. These differences should be considered as potential confounders in outcome analyses. *p < 0.05; **p < 0.01. MWU: Mann-Whitney U test; FE: two-tailed Fisher's exact test; IQR: interquartile range

Characteristic	Intervention (n = 60)	Control (n = 60)	P-value
Age (years)			
Median (IQR)	67.5 (53.0-74.0)	66.5 (59.0-74.0)	0.879 (MWU)
Sex, n (%)			
Male	21 (35.0%)	24 (40.0%)	0.706 (χ^2^)
Female	39 (65.0%)	36 (60.0%)	
Pain duration, n (%)			
6-12 months	14 (23.3%)	23 (38.3%)	0.114 (χ^2^)
>12 months	46 (76.7%)	37 (61.7%)	
Primary diagnosis, n (%)			
Disc herniation/discopathy	38 (63.3%)	49 (81.7%)	0.131 (χ^2^)
Muscle strain	11 (18.3%)	7 (11.7%)	
Migraine	7 (11.7%)	4 (6.7%)	
Crohn's disease	2 (3.3%)	0 (0.0%)	
Obesity	2 (3.3%)	0 (0.0%)	
Pain location, n (%)			
Lumbar (low back pain)	24 (40.0%)	41 (68.3%)	0.016* (χ^2^)
Sciatica	16 (26.7%)	8 (13.3%)	
Cervicalgia	9 (15.0%)	7 (11.7%)	
Cephalgia	7 (11.7%)	4 (6.7%)	
Abdominal/gastroesophageal reflux pain	4 (6.7%)	0 (0.0%)	
Pharmacological regimen, n (%)			
Monotherapy	31 (51.7%)	39 (65.0%)	0.195 (X^2^)
Combination therapy	29 (48.3%)	21 (35.0%)	
Medications used, n (%) (Fisher exact per drug)			
Paracetamol	38 (63.3%)	33 (55.0%)	0.458 (FE)
Ibuprofen	11 (18.3%)	13 (21.7%)	0.820 (FE)
Diclofenac	11 (18.3%)	5 (8.3%)	0.178 (FE)
Tramadol	5 (8.3%)	7 (11.7%)	0.762 (FE)
Pregabalin	8 (13.3%)	0 (0.0%)	0.006** (FE)
Paracetamol/codeine	2 (3.3%)	13 (21.7%)	0.004** (FE)
Lidocaine patch	0 (0.0%)	5 (8.3%)	0.057 (FE)
Other agents	15 (25.0%)	5 (8.3%)	0.026* (FE)

Age did not differ significantly between groups (Mann-Whitney U test, p = 0.879), and sex distribution was similar (chi-squared test, p = 0.706). Pain duration and primary diagnosis also did not differ significantly, although expected cell frequencies were low for some diagnostic categories. Pain location differed between groups (chi-squared test, p = 0.016), with lumbar pain being more common in the control group and sciatica, cervicalgia, and abdominal/gastroesophageal pain being relatively more frequent in the intervention group. Medication exposure also showed imbalances at the individual drug level. Pregabalin was prescribed only in the intervention group (13.3% vs. 0.0%; Fisher's exact test p = 0.006), paracetamol/codeine was more common in the control group (21.7% vs. 3.3%; Fisher's exact test p = 0.004), and other agents were more frequent in the intervention group (25.0% vs. 8.3%; Fisher's exact test p = 0.026).

**Figure 1:**
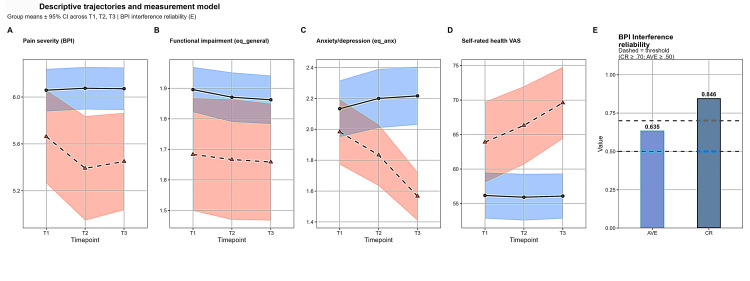
Descriptive longitudinal trajectories and BPI interference measurement model adequacy Sub-panels A through D display group means with 95% confidence intervals across the three assessment timepoints (T1 = baseline; T2 = mid-intervention; T3 = post-intervention) for the following: (A) pain severity, assessed as the BPI four-item composite (0-10; higher = greater severity); (B) functional impairment (EQ-5D eq_general; higher = greater impairment); (C) anxiety and depression (EQ-5D eq_anx; higher = greater impairment); and (D) self-rated global health (EQ-5D VAS; 0-100; higher = better health). Blue solid lines with filled circles represent the control group (waitlist; n = 60); red dashed lines with triangles represent the intervention group (expressive writing; n = 60). Shaded ribbons are 95% confidence intervals. Sub-panel E shows composite reliability (CR = 0.846) and average variance extracted (AVE = 0.635) for the BPI interference subscale. Dashed horizontal lines mark conventional adequacy thresholds (CR ≥0.70; AVE ≥0.50). BPI: Brief Pain Inventory; EQ-5D: EuroQol 5 Dimension; VAS: visual analogue scale This figure was created using R (version 4.4.0) (R Foundation for Statistical Computing, Vienna, Austria).

The control group had slightly higher mean pain severity (6.06, SD 0.72) than the intervention group (5.66, SD 1.57), and the intervention group had higher mean medication relief (56.0% vs. 50.0%), higher self-rated health (63.9 vs. 56.2 visual analogue scale (VAS) points), and slightly lower anxiety/depression (1.98 vs. 2.13). Across the observation period, control participants showed little change: pain severity remained close to 6.1, anxiety/depression increased slightly from 2.13 to 2.22, and health VAS remained near 56 points. Intervention participants showed a progressive decline in anxiety/depression (1.98, 1.83, 1.57) and a progressive rise in health VAS (63.9, 66.3, 69.6), while pain severity and functional impairment were broadly stable (Figure 1).

Pain severity as a longitudinal predictor of quality of life

**Figure 2:**
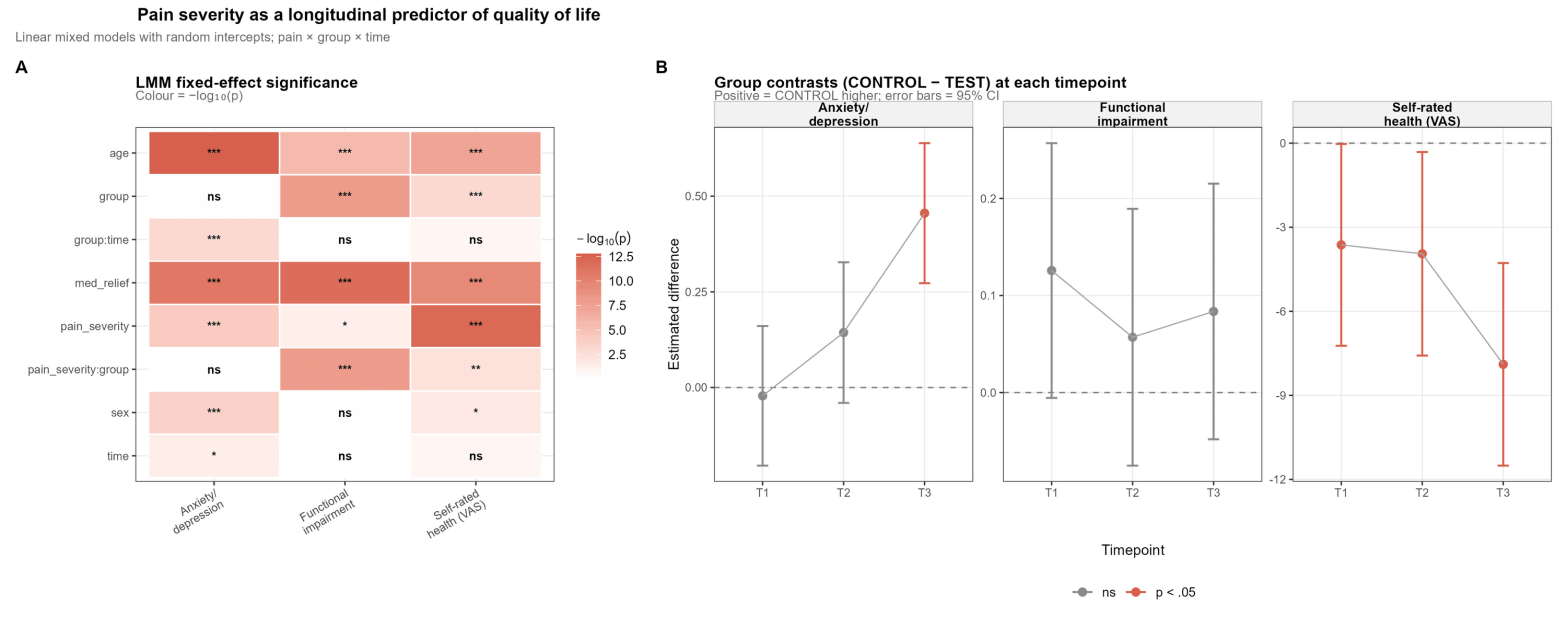
Pain severity as a longitudinal predictor of quality of life Three separate LMMs were fitted, one per outcome (anxiety/depression, functional impairment, self-rated health VAS), each including pain severity, group, time, their interactions, medication effectiveness, age, and sex as fixed effects, with random intercepts for subject. Sub-panel A presents a heatmap of fixed-effect significance across all three models; tile colour encodes −log₁₀(p), with deeper red indicating stronger evidence against the null. Age and medication effectiveness were consistently significant predictors. The pain severity × group interaction reached significance for functional impairment (p < 0.001) and self-rated health VAS (p < 0.01). Sub-panel B shows estimated marginal mean contrasts (control minus intervention) at each timepoint; positive values indicate control > intervention. Red points indicate p < 0.05; grey indicates non-significant contrasts. The gap in self-rated health between groups widened progressively from T1 to T3. LMMs: linear mixed models; VAS: visual analogue scale This figure was created using R (version 4.4.0) (R Foundation for Statistical Computing, Vienna, Austria).

Subsequently, the extent to which participant's pain severity predicted functioning in terms of daily life, anxiety, and perceived health was explored (Figure 2).

Higher pain severity was associated with greater functional impairment overall, but this relationship was significantly stronger in the intervention group than in the control group. This means that within the intervention group, pain and functional difficulty were more tightly coupled, namely, participants who experienced more pain also tended to experience proportionally greater difficulty with daily activities. In the control group, pain severity was only weakly linked to functional impairment. The members of both groups had undergone physical therapy in the past with no substantial results. Consequently, they presented again to the pain clinic of the University General Hospital of Alexandroupolis. Medication effectiveness was a consistently strong protective factor. Specifically, participants who perceived their medication as more effective reported lower functional impairment, regardless of group. Pain severity was a significant predictor of anxiety across both groups, but critically, the two groups diverged in their anxiety trajectories over time. At baseline, anxiety levels did not differ significantly between groups. By the final assessment, however, the control group showed substantially higher anxiety than the intervention group. This divergence, rather than a baseline difference, suggests that the intervention had a protective effect on anxiety that emerged progressively and was not simply a reflection of the groups starting at different points. The intervention group reported higher self-rated health than the control group at all three timepoints, and the gap between the groups widened over the course of the study. At baseline, the difference was approximately 4 points on the 0-100 scale; by T3, it had grown to approximately 8 points. Pain severity was the strongest single predictor of self-rated health, and again, the relationship between pain and self-rated health differed significantly by group, suggesting that pain had a steeper negative association with health perception in the intervention group.

Correlation structure of pain and quality of life

**Figure 3:**
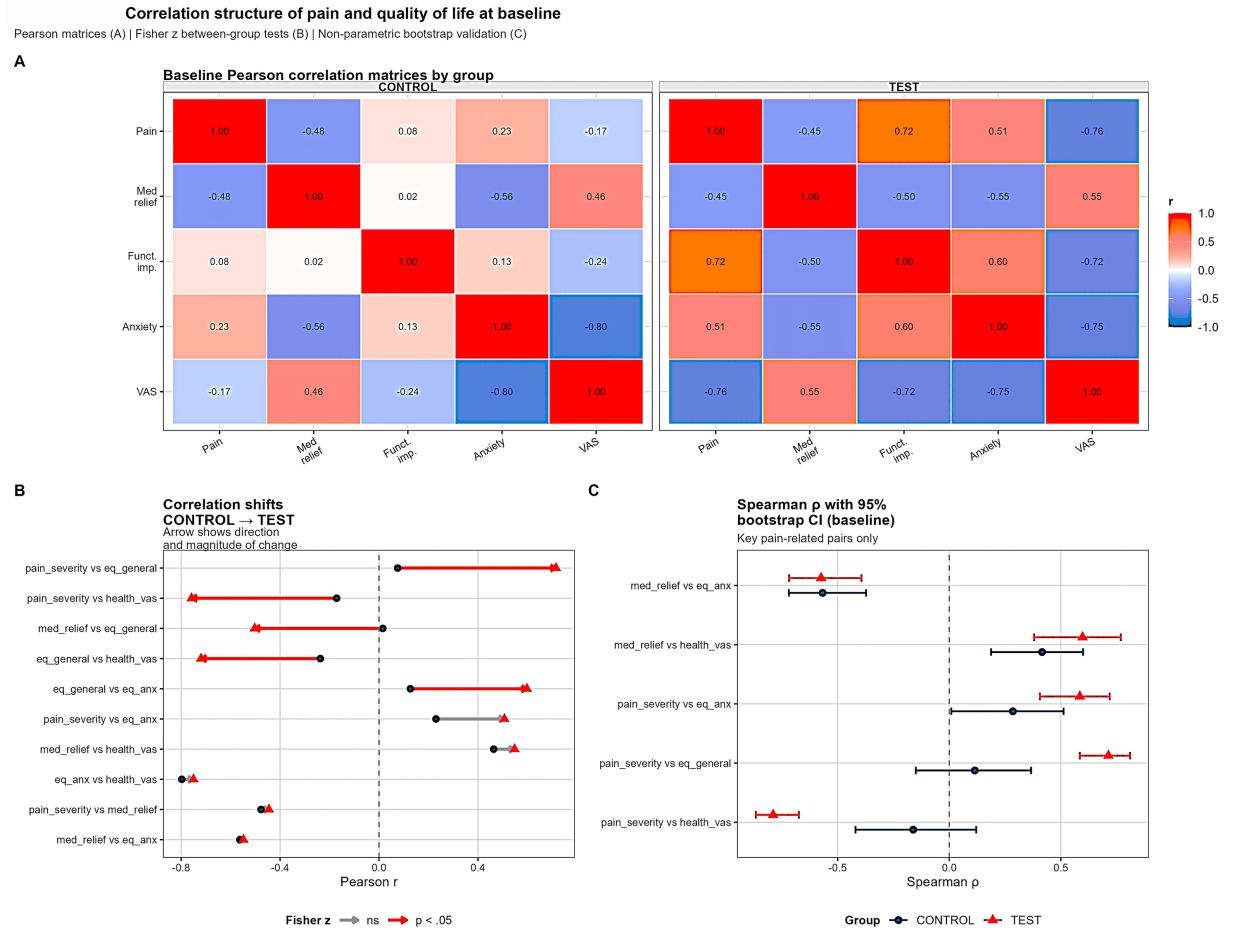
Baseline correlation structure of pain severity and quality of life by group Sub-panel A presents full 5 × 5 Pearson correlation matrices at baseline (T1) for the control and intervention groups across pain severity, medication effectiveness, functional impairment, anxiety/depression, and self-rated health VAS. Tile colour encodes Pearson r on a diverging palette (blue = negative; white = zero; red = positive). The correlation structure is markedly stronger and more coherent in the intervention group: pain severity correlates with functional impairment (r = 0.72 vs. r = 0.08), anxiety/depression (r = 0.51 vs. r = 0.23), and self-rated health VAS (r = −0.76 vs. r = −0.17). Sub-panel B displays directional arrows summarizing between-group differences for all 10 variable pairs; red arrows indicate a significant Fisher z-test (p < 0.05). Sub-panel C presents Spearman correlations with 95% bootstrap confidence intervals (1,000 replications) for the five variable pairs most relevant to the pain-quality of life hypothesis, confirming that the between-group differences are robust to distributional assumptions. VAS: visual analogue scale This figure was created using R (version 4.4.0) (R Foundation for Statistical Computing, Vienna, Austria).

Beyond asking whether pain predicts outcomes in a model, this analysis examined how all the key variables related to one another within each group, essentially mapping the internal structure of pain and quality of life (Figure 3).

In the control group, pain severity had weak or near-zero associations with functional impairment (r = 0.08), self-rated health (r = −0.17), and anxiety (r = 0.23) at baseline. Pain, in other words, was largely decoupled from the rest of the quality of life network in these participants. In the intervention group, the same correlations were dramatically larger, as pain severity was strongly associated with functional impairment (r = 0.72), self-rated health (r = −0.76), and anxiety (r = 0.51). Within the intervention group, pain was tightly woven into participants' overall health experience. Higher pain meant meaningfully worse functioning across the board. Notably, by the post-intervention assessment (T3), several of these correlations in the intervention group had attenuated: the pain-to-anxiety correlation decreased from 0.51 at baseline to 0.31 and the pain-to-health correlation from −0.76 to −0.61. This suggests that the expressive writing intervention may have progressively weakened the link between pain intensity and emotional and global health outcomes, meaning participants in the intervention group became to some extent less affected emotionally and perceptually by their pain over time. Formal tests (Fisher z-tests) confirmed that four of the correlation differences were statistically significant (all p ≤ 0.003). Non-parametric Spearman correlations with bootstrap confidence intervals corroborated the same pattern, confirming that the differences were not artefacts of distributional assumptions.

Moderation of pain effects by medication effectiveness

**Figure 4:**
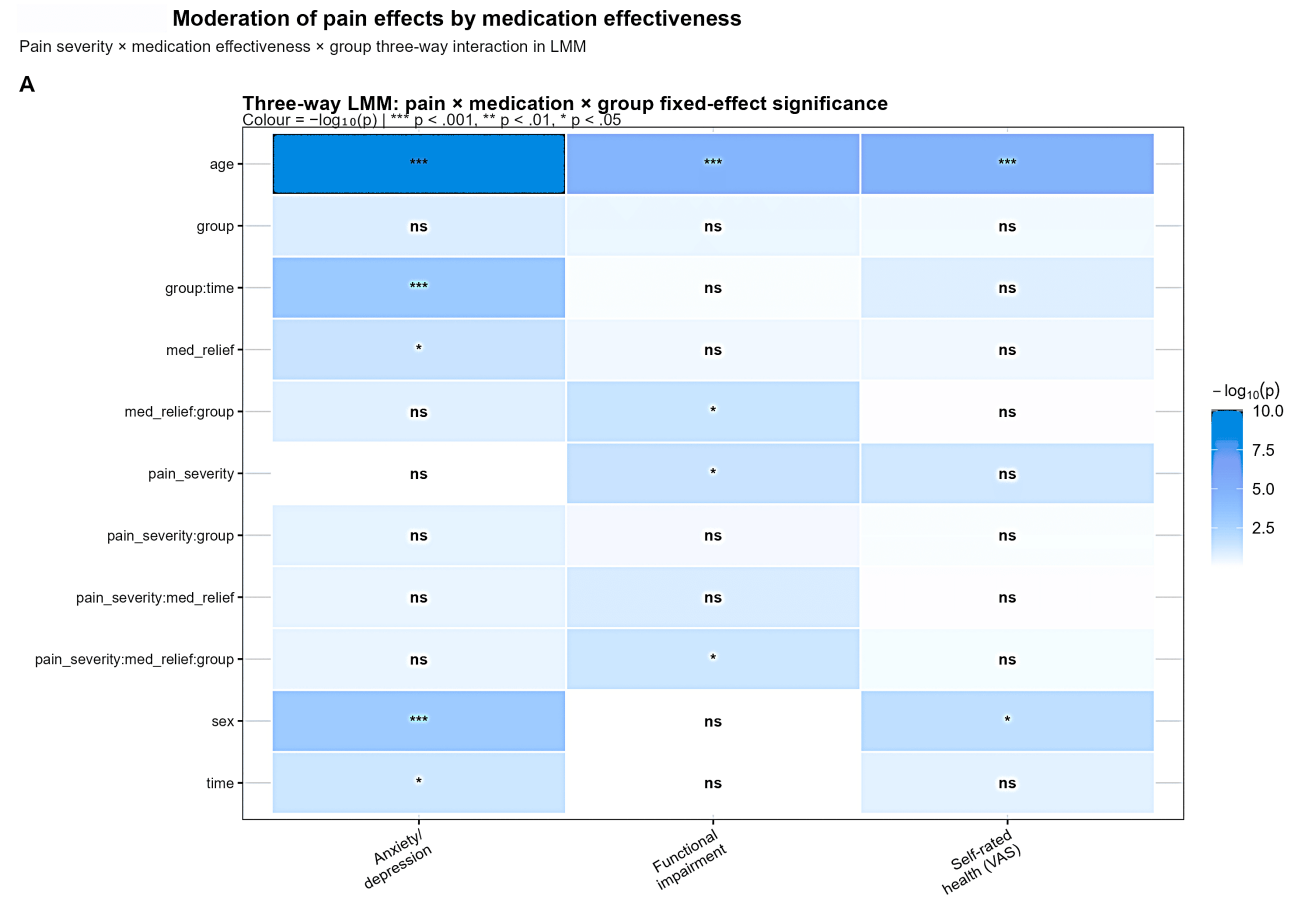
Three-way moderation of the pain-quality of life relationship by medication effectiveness and group Sub-panel A displays a heatmap of F-statistic significance for fixed effects across three LMMs, one per outcome. Each model specified the fully saturated three-way interaction of pain severity × medication effectiveness × group together with all constituent lower-order terms, time, sex, and age as covariates, and a random intercept for subject. The three-way interaction term was significant only for functional impairment (p < 0.05), indicating that the buffering effect of medication effectiveness on pain-related impairment differed between groups. Age was a robust predictor across all three outcomes. LMMs: linear mixed models; VAS: visual analogue scale This figure was created using R (version 4.4.0) (R Foundation for Statistical Computing, Vienna, Austria).

Next, the moderating effect of medication efficacy on the association between pain severity and quality of life, and whether this effect is contingent upon the intervention group, was investigated (Figure 4).

A significant three-way interaction (p = 0.044) indicated that the combination of pain severity and medication effectiveness predicted functional impairment differently in the intervention group versus the control group. In practical terms, the extent to which better medication effectiveness buffered against functional impairment differed by intervention status. Older participants tended toward lower functional impairment regardless of other factors; consequently, age was again a strong predictor. The three-way interaction was not significant for anxiety or self-rated health, indicating that medication effectiveness did not differentially moderate the pain-outcome relationship for these two outcomes across groups. However, the group-by-time interaction for anxiety remained significant (p < 0.001), reaffirming that anxiety trajectories diverged over time regardless of medication-related moderation. Medication effectiveness was a significant predictor in its own right for anxiety, and age and sex were significant for all three outcomes.

Mediation of pain-to-impairment pathway via anxiety

**Figure 5:**
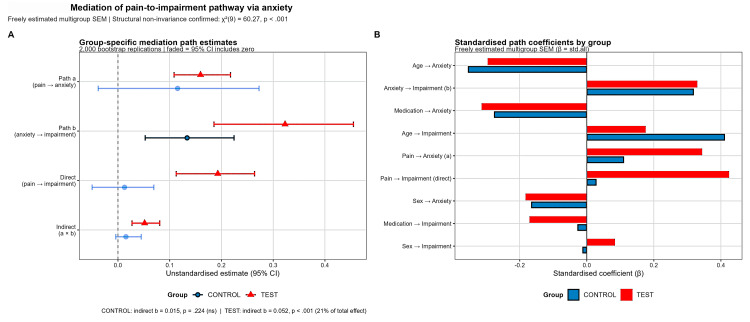
Multigroup structural equation model: mediation of the pain-to-impairment pathway via anxiety/depression A freely estimated multigroup path model was fitted simultaneously for both groups, with pain severity as the exogenous predictor, functional impairment as the distal outcome, and anxiety/depression as the mediator. Medication effectiveness, age, and sex were included as covariates. Structural non-invariance across groups was confirmed by a likelihood-ratio test (χ²(9) = 60.27; p < 0.001). Sub-panel A shows group-specific path estimates with 95% bootstrap confidence intervals (2,000 replications). In the control group, the mediation pathway was absent: path a (pain → anxiety) was non-significant (b = 0.115; p = 0.146) and the indirect effect did not differ from zero. In the intervention group, all paths were significant and the indirect effect accounted for 21.1% of the total effect (b = 0.052; 95% CI: 0.027, 0.081). Sub-panel B displays standardized path coefficients for both groups as a side-by-side bar chart, illustrating the qualitatively different structural organization between the two groups. SEM: structural equation modeling This figure was created using R (version 4.4.0) (R Foundation for Statistical Computing, Vienna, Austria).

This analysis tested whether anxiety mediates the link between pain and functional impairment. Specifically, it examined whether pain first increases anxiety, which in turn worsens daily functioning, and, critically, whether this pathway functions the same way in both groups (Figure 5).

The pathway structure was significantly different between the two groups (χ²(9) = 60.27; p < 0.001). Forcing the two groups to share the same pathway produced a model that fit the data poorly. Each group was therefore examined separately. In the control group, there was effectively no established pathway from pain to impairment, mediated or otherwise. Pain severity was not significantly associated with anxiety (the first step of the pathway), and the overall pain-to-impairment effect was non-significant. Anxiety did relate to functional impairment when pain was held constant, but because the pain-to-anxiety step was absent, the mediated route could not operate. Pain and quality of life, in this group, were structurally disconnected. In the intervention group, the full pathway was clearly established. Pain severity predicted anxiety (a significant association more than double the magnitude seen in the control group). Anxiety in turn predicted functional impairment, with a coefficient more than twice as large as in the control group. Pain also had a significant direct effect on functional impairment independent of anxiety. Together, anxiety accounted for approximately 21% of the total effect of pain on functional impairment, with the remaining 79% being a direct effect. This is partial mediation; anxiety is a meaningful part of the story, but not the whole story. Regardless of group, two factors were consistently protective against anxiety: first, higher perceived medication effectiveness, and second, older age. Female participants also reported lower anxiety in both groups. These findings are robust across model specifications.

Individual response patterns

**Figure 6:**
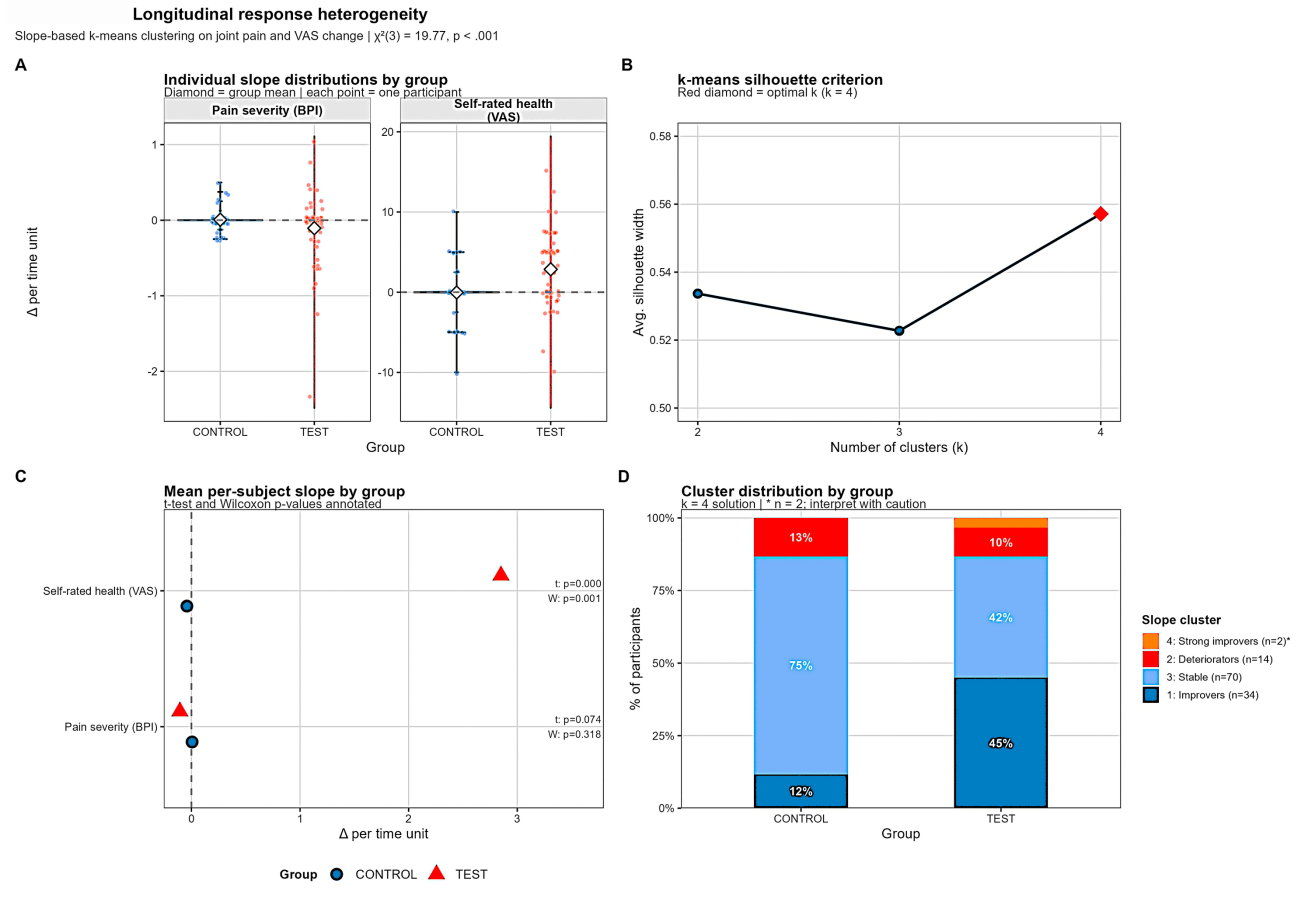
Longitudinal response heterogeneity: individual trajectory slopes and k-means cluster analysis Per-subject ordinary least squares slopes were estimated from within-person regression of each outcome on time, yielding one slope per participant per outcome. Sub-panel A displays violin plots with overlaid individual data points for pain severity and self-rated health VAS slopes by group. Sub-panel B shows average silhouette width for k = 2 to 4; the selected solution (k = 4; silhouette = 0.557) is marked. Sub-panel C plots group mean slopes with annotated test statistics; the intervention group showed a significantly steeper mean VAS slope (M = +2.85 vs. −0.04; t(118) = −4.26; p < 0.001). Sub-panel D presents stacked bar charts of cluster membership by group. Cluster membership differed significantly between groups (χ²(3) = 19.77; p < 0.001): 45% of intervention participants fell in the improvers cluster (declining pain, rising VAS) compared with 12% of control participants. VAS: visual analogue scale; BPI: Brief Pain Inventory This figure was created using R (version 4.4.0) (R Foundation for Statistical Computing, Vienna, Austria).

Group average effects can conceal important variation between individuals. Some people may improve substantially, while others stay the same or worsen. The trajectories of each individual were recorded. This analysis examined individual change trajectories, one slope per person, to understand the distribution and clustering of responses (Figure 6).

The average group trajectories confirmed that neither group changed significantly in pain severity over time. However, the intervention group showed a significantly steeper upward trajectory in self-rated health on an individual basis (average increase of approximately 2.9 VAS points per time unit vs. −0.04 in the control group; p < 0.001). This individual-level analysis detected a signal that the group-level model came close to but did not fully capture. Clustering participants by their combined pain and health trajectories identified four groups, with the four-cluster solution providing the best statistical separation. First were improvers (n = 34): declining pain severity and rising self-rated health. This was the profile most strongly associated with the intervention. Second were deteriorators (n = 14): rising pain and declining health, a pattern of worsening over time, distributed relatively evenly between groups. Third were stable (n = 70): minimal change in either direction, the most common profile overall. Fourth were strong improvers (n = 2): very large improvements in both outcomes. This cluster should be interpreted cautiously given the very small number of participants. The distribution across these clusters differed significantly by group (p < 0.001). In the intervention group, 45% of participants were classified as improvers, compared with only 12% of control participants. Conversely, 75% of control participants were classified as stable, compared with 42% of intervention participants. The intervention was therefore associated with a meaningful shift in the proportion of people who showed combined improvements in both pain and health, not just a shift in the average.

Pre-post change within each group (T1 to T3)

**Figure 7:**
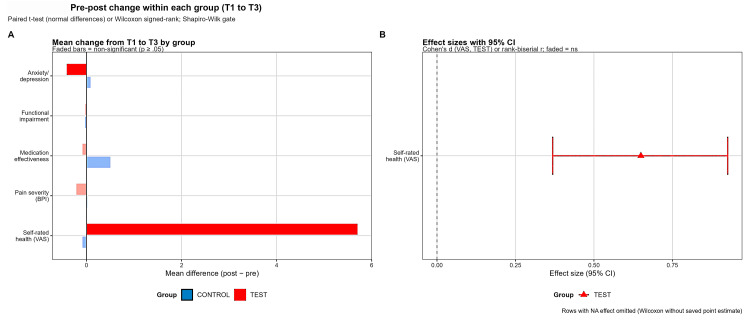
Pre-to-post change within each group from T1 to T3 Sub-panel A displays raw mean difference scores (T3 minus T1) for anxiety/depression, functional impairment, medication effectiveness, pain severity, and self-rated health VAS, shown separately by group. For impairment-coded outcomes, negative values represent improvement; for health-coded outcomes, positive values represent improvement. Faded bars indicate non-significant change. Shapiro-Wilk tests were applied to difference scores; paired t-tests were used where normality was not rejected and Wilcoxon signed-rank tests were applied otherwise. The only statistically significant pre-to-post change was an increase in self-rated health VAS in the intervention group (mean change = +5.67 VAS units; p < 0.001). Sub-panel B presents effect sizes with 95% confidence intervals: Cohen's d for the intervention group VAS change (d = 0.650; 95% CI: 0.369, 0.926) and rank-biserial correlations for all other combinations. VAS: visual analogue scale; BPI: Brief Pain Inventory This figure was created using R (version 4.4.0) (R Foundation for Statistical Computing, Vienna, Austria).

This analysis directly compared each participant's scores at the end of the study (T3) with their scores at the start (T1), asking whether change was statistically significant within each group (Figure 7).

In the control group, none of the five primary outcomes showed a statistically significant change from T1 to T3. Pain remained essentially flat, medication effectiveness was stable, functional impairment showed a marginal non-significant improvement, anxiety edged slightly upward, and self-rated health was unchanged. This is consistent with the expectation that the passage of time alone, without an active intervention, would not produce meaningful change in chronic pain patients. In the intervention group, two outcomes improved significantly between baseline and post-intervention. Anxiety and depression (eq_anx) decreased by an average of 0.42 points on the 1-3 scale (p < 0.001), with a large effect size. This represents a clinically meaningful reduction in the anxiety and depression burden reported by participants following the creative writing intervention. Self-rated health (VAS) increased by an average of 5.7 points on the 0-100 scale (p < 0.001; Cohen's d = 0.65; 95% CI: 0.37, 0.93), a moderate-to-large effect. Participants rated their overall health substantially better at post-intervention than at baseline. Pain severity showed a non-significant trend toward improvement in the intervention group (average reduction of 0.21 points), and a supplementary analysis of individual EQ-5D found that pain interference with daily activities decreased significantly in the intervention group (p < 0.001), suggesting that while the intensity of pain did not change significantly, its impact on daily life did.

Group and time effects across all primary outcomes

**Figure 8:**
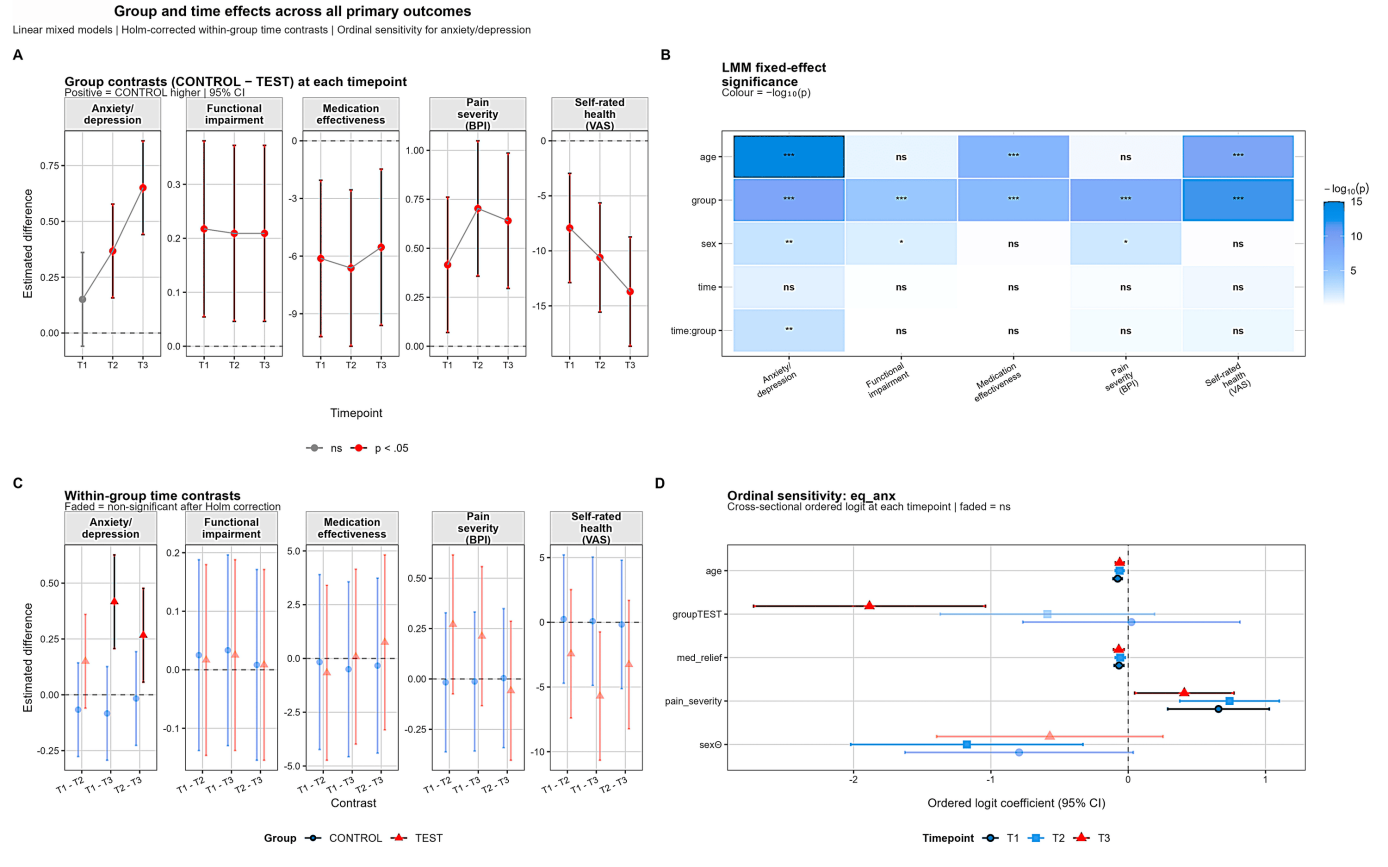
Between-group and within-group differences across all primary outcomes over the observation period Five separate LMMs were fitted (one per outcome), each specifying group, time, the group × time interaction, age, and sex as fixed effects and a random intercept for subject. Sub-panel A shows estimated marginal mean contrasts (control minus intervention) at each timepoint; red points indicate p < 0.05. Persistent between-group differences were observed for anxiety/depression, functional impairment, pain severity, and self-rated health VAS. Sub-panel B presents a heatmap of fixed-effect F-statistic significance; group was significant for all five outcomes (all p < 0.001) and the group × time interaction was significant only for anxiety/depression (p = 0.008). Sub-panel C presents pairwise within-group time contrasts (Holm-corrected); significant within-group change was limited, with the most notable changes observed for anxiety/depression in the intervention group between T1 and T3. Sub-panel D presents an ordinal logistic sensitivity analysis for anxiety/depression fitted independently at each timepoint, confirming that the primary LMM findings were robust to distributional assumptions. LMMs: linear mixed models; VAS: visual analogue scale This figure was created using R (version 4.4.0) (R Foundation for Statistical Computing, Vienna, Austria).

This final analysis provided the most comprehensive view. All five outcomes were examined simultaneously across all three timepoints to determine how group, time, and their interaction explained the data without conditioning on pain severity as a predictor (Figure 8).

The intervention group reported lower mean pain severity than the control group at all three timepoints (T1: 0.42 points lower; T2: 0.70 points lower; T3: 0.64 points lower), but neither group changed meaningfully in pain severity over time. The intervention did not reduce pain at the group level. It improved how people lived with their pain. The intervention group reported consistently higher perceived medication effectiveness throughout (approximately 5-7 percentage points higher at each timepoint). This difference was stable and did not change over time, suggesting it reflects a baseline characteristic of the groups rather than an intervention effect on medication perception. The control group showed consistently higher functional impairment than the intervention group at all three timepoints, with a difference of approximately 0.21 points (on a 0-3 scale) that remained constant across the observation period. Neither group showed significant within-group change in functional impairment. This was the outcome with the most interesting temporal pattern. The two groups did not differ significantly in anxiety at baseline, but diverged progressively and significantly over the observation period. By T2, the gap was already significant (control 0.37 points higher than intervention; p < 0.001), and by T3, it had widened further (control 0.65 points higher; p < 0.001). Within the intervention group, anxiety decreased significantly between T1 and T3 (estimate = 0.42 points; p < 0.001) and between T2 and T3 (estimate = 0.27 points; p = 0.027). In the control group, no significant within-group change occurred at any interval. This pattern is important because it suggests that the intervention's effect on anxiety was not immediate but accumulated over time, manifesting most clearly in the later phase of the observation period. The cross-sectional ordinal logistic models at each timepoint confirmed that the intervention group effect on anxiety was non-significant at T1 and T2 but highly significant at T3 (p < 0.001), corroborating the LMM result through an entirely different statistical method. The intervention group reported substantially better self-rated health than the control group at all timepoints, and the advantage grew over time, namely, approximately 8 VAS points at T1, 11 points at T2, and 14 points at T3. This widening gap, combined with the finding of a significant within-group improvement, indicates that the intervention was associated with a progressive enhancement of participants' perception of their overall health.

## Discussion

In this non-randomized study of 120 patients with chronic pain, creative writing intervention was associated with significant reductions in anxiety and depression and meaningful improvements in self-rated health by the end of the observation period. Pain severity itself did not change significantly at the group level, but the way pain related to other aspects of quality of life differed markedly between the two groups.

The moderating role of medication effectiveness on the pain-impairment relationship was specific to functional impairment and was contingent on group membership. The participants in both groups had previously undergone physical therapy with no significant results. This was indeed the reason they presented again to the pain clinic of the University General Hospital of Alexandroupolis. This means the intervention changed not just mean levels of functioning but the mechanism by which medication effectiveness relates to pain and impairment. The creative writing intervention was associated not just with better outcomes but with a qualitatively different organizational structure of the pain-anxiety-impairment relationship. In the control group, this chain was effectively absent. In the intervention group, it was active, with anxiety serving as a partial mediator, suggesting that the intervention may have enabled participants to engage more consciously and meaningfully with how pain affects their emotional and functional life, rather than experiencing these as disconnected. The intervention did not produce uniform improvement across participants. However, it substantially increased the proportion of individuals showing a pattern of combined pain reduction and health improvement, from 12% in the control group to 45% in the intervention group. The majority of control participants simply stayed the same. Also, the intervention produced statistically significant and clinically meaningful reductions in anxiety/depression and improvements in self-rated health between baseline and post-intervention. The control group showed no significant change in any outcome over the same period. Across all five outcomes and all timepoints, the intervention group consistently showed better health profiles. The most striking finding is the progressive and late-emerging divergence in anxiety, absent at baseline, significant by mid-point, and substantial by post-intervention. This temporal pattern suggests an accumulating effect of the creative writing practice.

In a study by Sundaram et al. regarding the utilization of expressive writing in patients with chronic pain within primary healthcare settings, the Three-Minute Mental Makeover (3MMM) model was followed [18]. In the present study, creative writing was systematically utilized as an intervention method, and participants were asked to record the thoughts and feelings they experienced during their treatment for 15 minutes daily over a period of two weeks. Based on these two studies and the research by Tian et al., it was indicated that the participants' psychological well-being and their perceived quality of life improved through the creative writing intervention [19]. In a study by Schaufel et al. regarding the long-term consequences of expressive writing, its impact was examined at intervals exceeding 12 months following the patients' treatment. In that specific study, participants included not only the patients themselves but also medical staff, doctors and nurses, as well as members of the patients' family environment. In their research, communication between researchers and participants was conducted via telephone or email, while in the present study, it took place in person [20]. Furthermore, according to a study by Gerger et al., writing-based interventions can significantly contribute to the reduction of post-traumatic stress disorder (PTSD) symptoms, even in the long term, since it functions as a beneficial distraction [21]. Based on the aforementioned studies, the present research, and a study by Troop et al., it can be inferred that patients gain a better understanding of their condition through creative writing and tend to adopt a positive outlook on life [22]. In this manner, patients with chronic pain can remain productive, according to our study and a study by Thomsen et al., while simultaneously reducing their healthcare utilization needs [23]. Engagement in creative writing, based on a Lee et al. study, causes no harm and has an undoubtedly positive effect, even in short-term interventions [24].

Regarding younger patients with chronic pain (college and university students), it has been observed in a Root and Nosek study that when prompted to discuss their condition through expressive writing, they typically utilize negatively charged expressions, reflecting their frustration with their state of health [25]. In our study, the median age was 67.5 for the intervention and 66.5 for the control group. Based on 21 out of 31 reviewed studies, a Porras-Segovia et al. study showed, as this study, that creative writing contributes significantly to the better management of depression [26]. The impact of creative writing has also been examined in individuals who have experienced trauma, a group estimated to reach 90% of the US population. A study by Glass et al. concluded that creative writing increases the resilience of these individuals and reduces depressive symptoms. In that case, the intervention lasted six weeks, whereas our study conducted a two-week intervention. Moreover, that study involved a general sample of individuals with traumatic experiences, rather than exclusively patients with chronic pain, as in our research. Nevertheless, both studies demonstrated that creative writing can have a positive effect on patients' psychological well-being, despite any pain or trauma they may have experienced [27]. Additionally, according to the study by Verhagen et al., writing-based interventions can bolster self-esteem and, consequently, contribute to the reduction of depressive symptoms [28].

The advantages of the present study include the direct, face-to-face communication with the patients, which established a bond of trust between the researcher and the participating patients. Moreover, a distinct advantage is that the texts and data produced by the patients were highly engaging and insightful, providing significant value to both their families and the attending medical staff. The relatively small number of participants and the limited duration of the intervention may be considered as limitations of the study. Additionally, the study was non-randomized according to the analysis documentation, and there were baseline differences in pain location, medication use, pain severity, medication relief, and self-rated health.

## Conclusions

Across all complementary analyses, a coherent and convergent picture emerges. The creative writing intervention did not cure pain; mean pain severity did not change significantly in either group across the observation period. What it did do was alter, in consistent and meaningful ways, how participants related to their pain, experienced their health, and functioned emotionally and in daily life. In addition, tailoring creative writing interventions to each patient's appropriate timing and respecting their privacy can enhance therapeutic benefits.

Anxiety and depression decreased significantly in the intervention group by post-intervention (p < 0.001), an effect that was not present at baseline and emerged progressively over the course of the study. Self-rated health improved significantly in the intervention group (an average of 5.7 VAS points from baseline to post-intervention; d = 0.65), with the group advantage over control widening at each successive timepoint. Pain interference with daily activities decreased significantly in the intervention group, even though overall pain severity did not change significantly, suggesting that participants functioned better despite their pain. The proportion of participants showing a combined pattern of declining pain and improving health was nearly four times higher in the intervention group (45%) than in the control group (12%). The relationship between pain and quality of life outcomes was structurally reorganized in the intervention group. Pain was more coherently integrated into the quality of life network, the anxiety-mediation pathway was active, and the coupling between pain intensity and emotional outcomes attenuated over time. Medication effectiveness, older age, and female sex were consistently protective factors against anxiety across all analyses and in both groups, highlighting that individual characteristics play an important independent role in pain-related psychological well-being.

Consequently, the utilization of creative writing in medical practice is deemed beneficial, particularly within the context of caring for patients with chronic pain. This is due to its positive impact on their psycho-emotional state and its capacity to facilitate the management of pain-related anxiety and depression. Its contribution, when combined with appropriate pharmacological treatment, can significantly improve the patients' quality of life, enabling them to remain active and productive. Creative writing interventions may be worth testing as low-risk adjuncts for chronic pain patients whose main burden includes anxiety/depression and poor perceived health. A future definitive trial should use concealed random allocation, stratification by pain location, potentially facilitated by patient wearable technology, and medication class, prespecified primary outcomes, and longer follow-up.

## Figures and Tables

**Figure 9:**
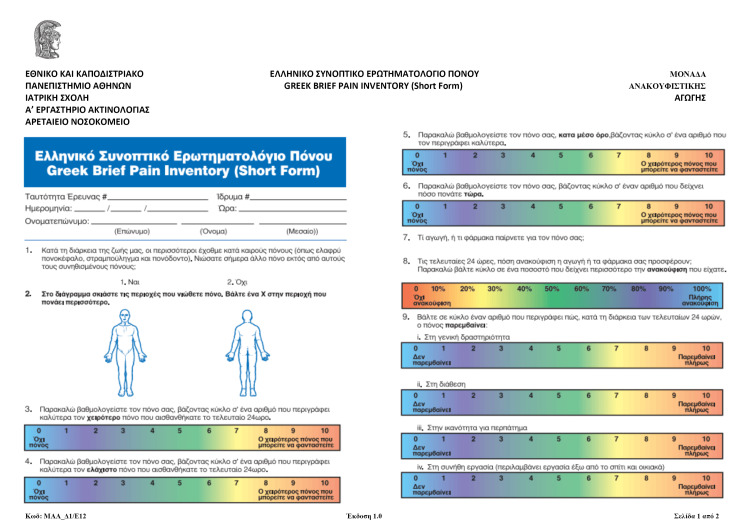
Greek Brief Pain Inventory (page 1)

**Figure 10:**
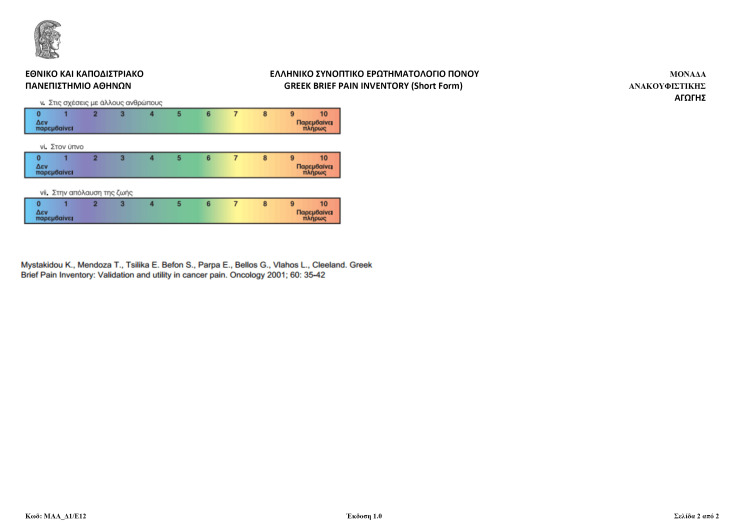
Greek Brief Pain Inventory (page 2)

**Figure 11:**
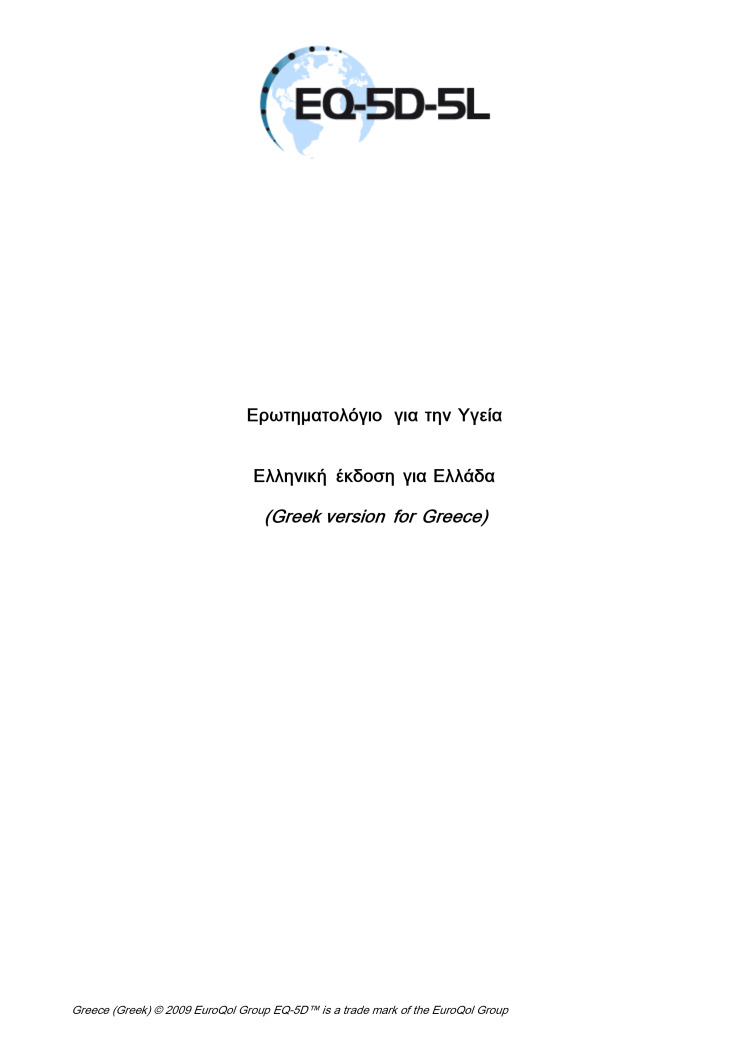
EuroQol 5 Dimension questionnaire Greek version (page 1)

**Figure 12:**
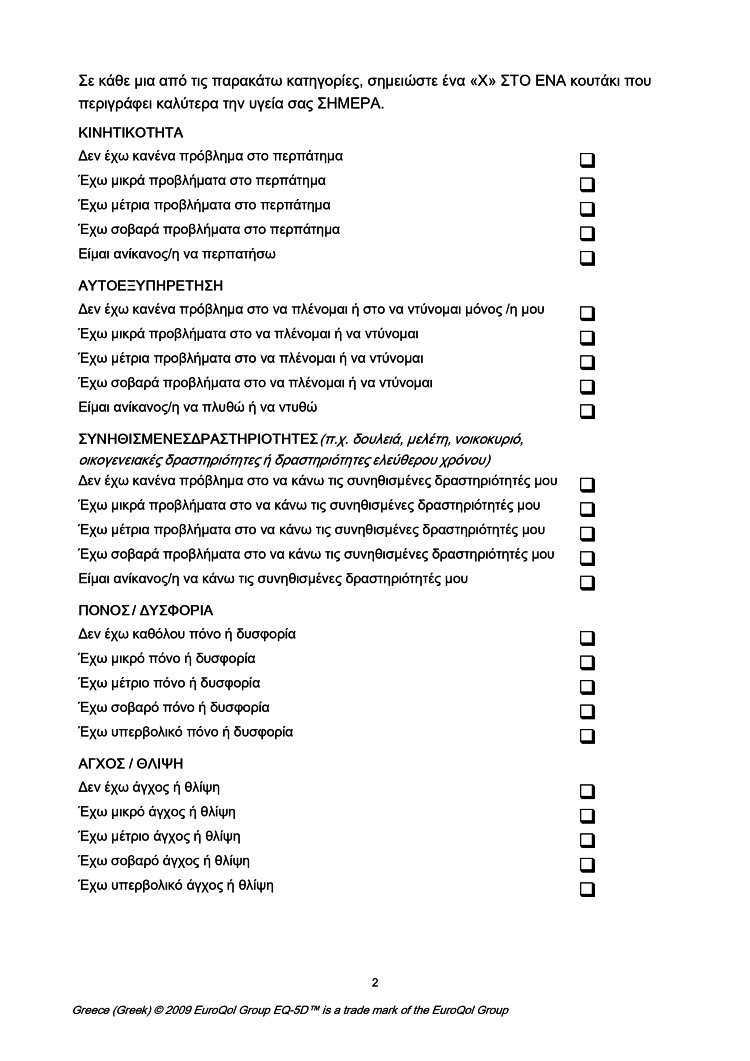
EuroQol 5 Dimension (page 2)

**Figure 13:**
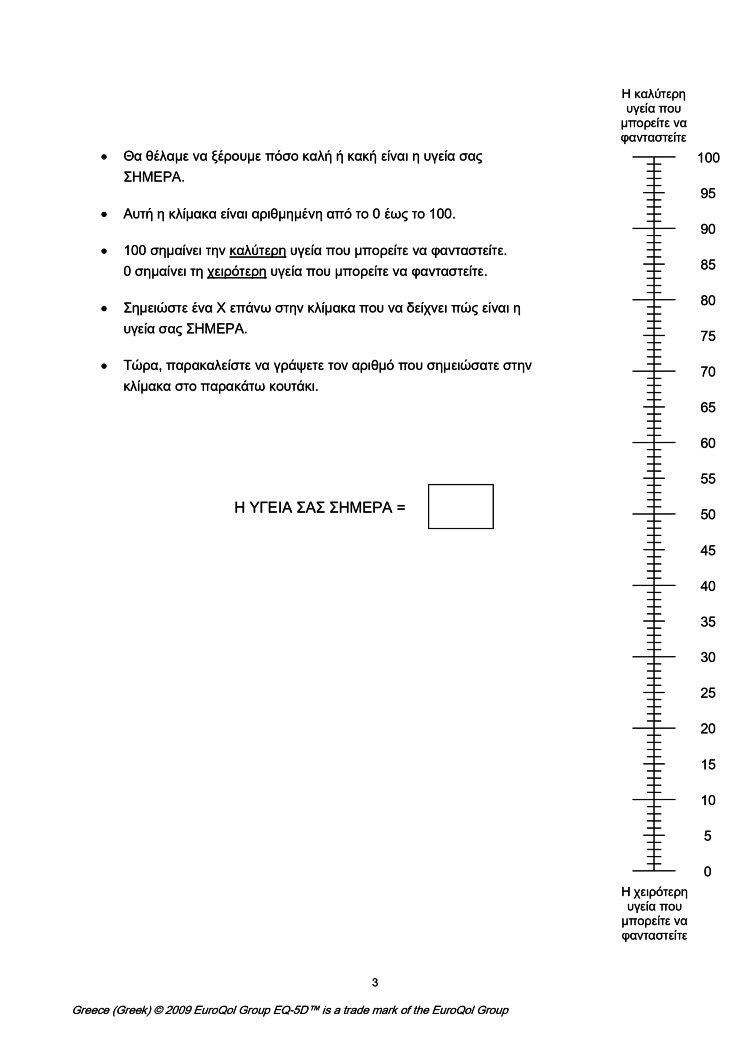
EuroQol 5 Dimension (page 3)

## Appendices

Appendix A

Appendix B

## References

[1] Nicholas M, Vlaeyen JW, Rief W (2019). The IASP classification of chronic pain for ICD-11: chronic primary pain. Pain.

[2] Breivik H, Collett B, Ventafridda V, Cohen R, Gallacher D (2006). Survey of chronic pain in Europe: prevalence, impact on daily life, and treatment. Eur J Pain.

[3] Bäckryd E, Alföldi P (2023). Chronic pain and its relationship with anxiety and depression [Article in Swedish]. Lakartidningen.

[4] McKillop AB, Carroll LJ, Dick BD, Battié MC (2018). Measuring participation in patients with chronic back pain-the 5-Item Pain Disability Index. Spine J.

[5] Shraim MA, Massé-Alarie H, Hall LM, Hodges PW (2020). Systematic review and synthesis of mechanism-based classification systems for pain experienced in the musculoskeletal system. Clin J Pain.

[6] Pentury HJ, Anggraeni AD, Pratama D (2020). Improving students 21st century skills through creative writing as a creative media. Deiksis.

[7] Adams K (2013). Expressive Writing: Foundations of Practice. https://www.abebooks.com/9781475803129/Expressive-Writing-Foundations-Practice-Easy-1475803125/plp.

[8] Moore M, Meekings S (2021). The Place and the Writer: International Intersections of Teacher Lore and Creative Writing Pedagogy. https://www.bloomsbury.com/uk/place-and-the-writer-9781350127173/.

[9] Wu J, Chen DV (2020). A systematic review of educational digital storytelling. Comput Educ.

[10] Zhang W, Jhang J, Greenwell MR (2023). Effects of replay and rehearsal expressive writing on mental health: a randomized controlled trial. J Ment Health.

[11] Guo L (2023). The delayed, durable effect of expressive writing on depression, anxiety and stress: a meta-analytic review of studies with long-term follow-ups. Br J Clin Psychol.

[12] Bullock O, McFarlane J (2025). Poetry and well-being: a pilot programme to evaluate the impact of creative writing for patients in short-term and long-term rehabilitation. Med Humanit.

[13] Smith MA, Hoult LM, Rippon D (2025). Facilitators and barriers to engaging in expressive writing among health and social care professionals. PLoS One.

[14] Pennebaker JW (2018). Expressive writing in psychological science. Perspect Psychol Sci.

[15] Mendoza T, Mayne T, Rublee D, Cleeland C (2006). Reliability and validity of a modified Brief Pain Inventory short form in patients with osteoarthritis. Eur J Pain.

[16] Ben-Shachar M, Lüdecke D, Makowski D (2020). effectsize: estimation of effect size indices and standardized parameters. J Open Source Softw.

[17] Lenth RV, Piaskowski J (2026). emmeans: estimated marginal means, aka least-squares means. https://cran.r-project.org/web/packages/emmeans/index.html.

[18] Sundaram CC, Thoele DG, Henningfield MF, Zaborek J, Hagen S (2024). Exploring expressive writing with patients with chronic pain during primary care visits. WMJ.

[19] Tian X, Zhou X, Sun M, Yu NX, Peng Y, Zheng X, Xiao W (2024). The effectiveness of positive psychological interventions for patients with cancer: a systematic review and meta-analysis. J Clin Nurs.

[20] Schaufel M, Moss D, Donovan R, Li Y, Thoele DG (2021). Better together: long-term behaviors and perspectives after a practitioner-family writing intervention in clinical practice. Perm J.

[21] Gerger H, Werner CP, Gaab J, Cuijpers P (2022). Comparative efficacy and acceptability of expressive writing treatments compared with psychotherapy, other writing treatments, and waiting list control for adult trauma survivors: a systematic review and network meta-analysis. Psychol Med.

[22] Troop NA, Chilcot J, Hutchings L, Varnaite G (2013). Expressive writing, self-criticism, and self-reassurance. Psychol Psychother.

[23] Thomsen AB, Sørensen J, Sjøgren P, Eriksen J (2002). Chronic non‐malignant pain patients and health economic consequences. Eur J Pain.

[24] Lee Y, Kim D, Lim JE (2023). Do expressive writing interventions have positive effects on Koreans?: a meta-analysis. Front Psychiatry.

[25] Root K, Nosek S (2023). Understanding how college students characterise and cope with chronic pain: a thematic analysis of expressive writing samples. Med Humanit.

[26] Porras-Segovia A, Escobedo-Aedo PJ, Carrillo de Albornoz CM (2024). Writing to keep on living: a systematic review and meta-analysis on creative writing therapy for the management of depression and suicidal ideation. Curr Psychiatry Rep.

[27] Glass O, Dreusicke M, Evans J, Bechard E, Wolever RQ (2019). Expressive writing to improve resilience to trauma: a clinical feasibility trial. Complement Ther Clin Pract.

[28] Verhagen RM, Carrico AW, Hylton EM, Stuetzle R, Ironson G (2023). Exploring self-esteem during expressive writing about trauma predicts decreased depression in people with HIV. AIDS Care.

